# A Method for Evaluating the Electro-Mechanical Characteristics of Piezoelectric Actuators during Motion

**DOI:** 10.3390/s120911559

**Published:** 2012-08-24

**Authors:** Tao Jin, Akihiro Takita, Mitra Djamal, Wenmei Hou, Hongzhi Jia, Yusaku Fujii

**Affiliations:** 1 Department of Electronic Engineering, Faculty of Engineering, Gunma University, 1-5-1 Tenjin-cho, Kiryu, Gunma 376-8515, Japan; E-Mails: takita@el.gunma-u.ac.jp (A.T.); fujii@el.gunma-u.ac.jp (Y.F.); 2 Department of Physics, Faculty of Mathematics and Natural Sciences, Institut Teknologi Bandung, Jalan Ganesa 10, Bandung 40132, Indonesia; E-Mail: mitra@fi.itb.ac.id; 3 Shanghai Key Laboratory of Modern Optical System, School of Optical-Electrical and Computer Engineering, University of Shanghai for Science and Technology, Shanghai 200093, China; E-Mail: houwenmei@vip.citiz.net (W.M.H.); jiahongzhi0715@sina.com (H.Z.J.)

**Keywords:** piezo actuator, Doppler frequency shift, dynamic measurement, interferometer

## Abstract

The electro-mechanical characteristics of piezoelectric actuators which have being driven are evaluated in this paper. The force generated by actuators is measured as an inertial force of a corner cub prism which is attached to the actuators. The Doppler frequency shift of a laser beam, due to the motion of actuator, is accurately measured by a heterodyne interferometer. Subsequently, the mechanical quantities, such as velocity, acceleration, force, power and displacement, are calculated from the Doppler frequency shift. With the measurement results of current and voltage of the actuator, the relationships between electrical and mechanical characteristics are evaluated.

## 1. Introduction

Piezo-electric (PZT) actuator, with high stiffness, compact size and high resolution, has been applied in many areas related to precision position and force control and/or measure [1–3]. The static output characteristics of conventional PZT actuator are typically evaluated by standard static methods, e.g., using static weights under static conditions. The dynamic outputs of PZT actuator including force and displacement are usually measured by using force and displacement transducer, respectively. However, so far, no standard dynamic calibration method for force transducer has been proposed. In other words, the uncertainly of transducer influences the accuracy of dynamic measuring results.

Instead of force transducer, a spring-mass-damper (SMD) is used for dynamic analysis characteristics of actuators [4,5]. The dynamic force is measured as the impedance interactions between actuators and load structure, *i.e.*, SMD. In this method, the stiffness of spring and the coefficient of piezoelectric thin film stocked in actuator during motion direction are considered constant. However, compound components such as piezo-stack actuators can not be described sufficiently by piezoelectric coefficients, which vary with temperature, pressure, electric field, mechanical and electrical boundary conditions *etc.* [6,7]. A mass was loaded to PZT actuator by using a rotating arm via a high stiffness transmission rod [8]. Several displacement transducers were used to measure the rotation of the arm in order to calculate the force generated or applied on PZT actuator. In this device, the accuracy of displacement measurement is sensitive to temperature. Some models for the dynamic analysis of the electro-mechanical characteristics of PZT actuator are proposed [9,10]. In those models, the design of mechanical parameters is based on testing actuator with dead weight, known structure or transducer *etc.* Several kinds of interferometer, such as signal frequency interferometer, laser Doppler interferometer and laser diode interferometer *etc.*, are introduced to accurately measure the displacement of actuators/piezoelectric thin film [11,12]. However, they failed to measure the dynamic force. Some studies, in which dynamic responses of force transducers against some typical dynamic forces, such as impact force [13], step force [14] and oscillation force [15], are mentioned based on the Levitation Mass Method (LMM). In the LMM, the inertial force of a mass, which is levitated using an aerostatic linear bearing with sufficiently small friction [16], is used. Acceleration of the mass is accurately measured by means of an optical interferometer [17]. The LMM has been applied to dynamic calibration for force transducers and material testers [18,19].

This paper aims to measure and evaluate the electric-mechanical characteristics of piezoelectric actuators during dynamic condition. The method proposed in this study does not need any force transducer and the characteristics of host or preloading. Our method, the modified LMM, is different from the conventional Doppler velocimeter. First, in the LMM, the inertial force is measured as the definition of force, the product of mass and acceleration. Second, the beat frequencies of the laser lights are highly accurately calculated by the Zero-crossing Fitting Method (ZFM), which has proposed by us [17]. All other quantities, e.g., velocity, acceleration, displacement and force, are calculated from the frequencies. In this paper, a corner-cube prism (CC) is attached to the tip of PZT actuators with high stiffness metal blocks. The force generated by the actuator is calculated as the inertial force of the total mass of CC and blocks. An optical interferometer is introduced to measure the Doppler frequency shift caused by the motion of PZT actuators. Velocity, acceleration, force, power and displacement of the actuators during motion are measured from the Doppler frequency. Force-displacement behaviors of the actuators under dynamic condition are characterized. The relationships of energy conversion between electrical and mechanical domains are also characterized based on the observed results.

## 2. Experimental Setup

**Figure 1.**
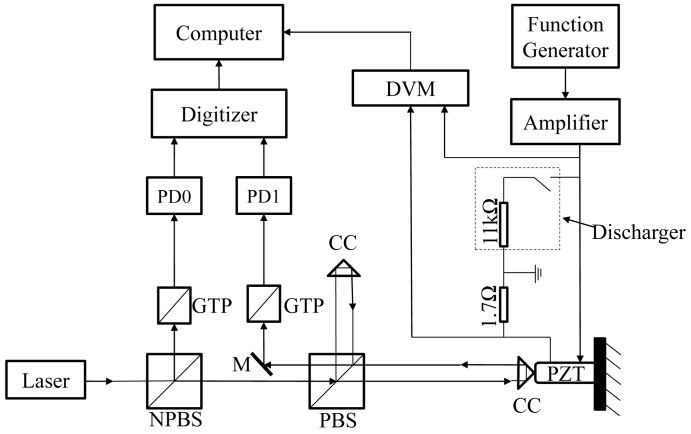
Experimental setup.

**Figure 2.**
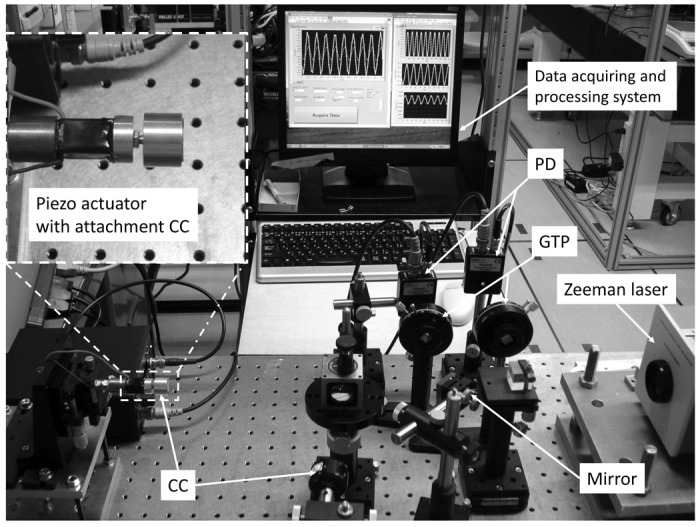
Photographs of measurement system.

A schematic diagram of experimental setup and photographs of measurement system are shown in Figures 1 and 2, respectively. A CC is attached to a piezo-stack actuator with some metal blocks and the total mass of metal blocks including the CC is 9.07*g*. The actuator is driven by a sinusoid signal at a frequency of 100*Hz* which is generated by a function generator and amplified by a piezoelectric driver. The actuator is connected with a shunt resistor. The voltage of actuator and shunt resister are measured by a digital voltmeter (DVM), which is based on a data acquisition card (PCI-6221, manufactured by National Instruments Corp., USA). The current of actuator is calculated as the current of shunt resistor. A discharger is used for discharging the actuator.

A Zeeman-type two-frequency He-Ne laser is used as the light source of the optical interferometer. The interferometer has two photo-detectors: PD0 and PD1. The waveforms appearing at PD0 and PD1 are recorded using a digitizer (model: 5102; manufactured by National Instruments Corp., USA) with the sampling rate of 20 MS/s and the sampling length of 5 m, then the frequencies are accurately calculated from the waveform using ZFM, in which all the zero-crossing are used for estimating the frequency in each measurement period [17].

The frequency difference, *f_rest_* between the two orthogonal polarization states light emitted from the laser is monitored using a Glan–Thompson prism (GTP) and PD0. The total force, *F*, action on the attached mass is calculated as the product of its mass *M* and acceleration *a* as follows:

F=Ma

In the measurement, *F* is considered to be generated by the actuator and applied to the mass at the contact surface between the actuator and the mass. The acceleration is calculated from the velocity of mass which is measured as the Doppler shift frequency, *f_Doppler_*.

$$\begin{array}{l} v=\lambda_{\text{air}}f_{\text{Doppler}}/2\\ f_{\text{Doppler}}=f_{\text{rest}}-f_{\text{beat}} \end{array}$$

where *λ_air_* is the wavelength of the signal beam under the experimental conditions, and *f_beat_* that appeared at PD1 is the beat frequency, which is the frequency difference between the signal beam and the reference beam. The direction of the coordinate system for the velocity, acceleration and force is toward the right in Figure 1. Its origin is set to be the center of movement of the actuator. All the other mechanical quantities, such as the position, acceleration and force of the mass, are numerically calculated from the velocity.

## 3. Results and Discussion

Three PZT actuators with difference size named as P1, P2 and P3 shown in Table 1 are tested by the above mentioned experiment setup.

**Figure 3.**
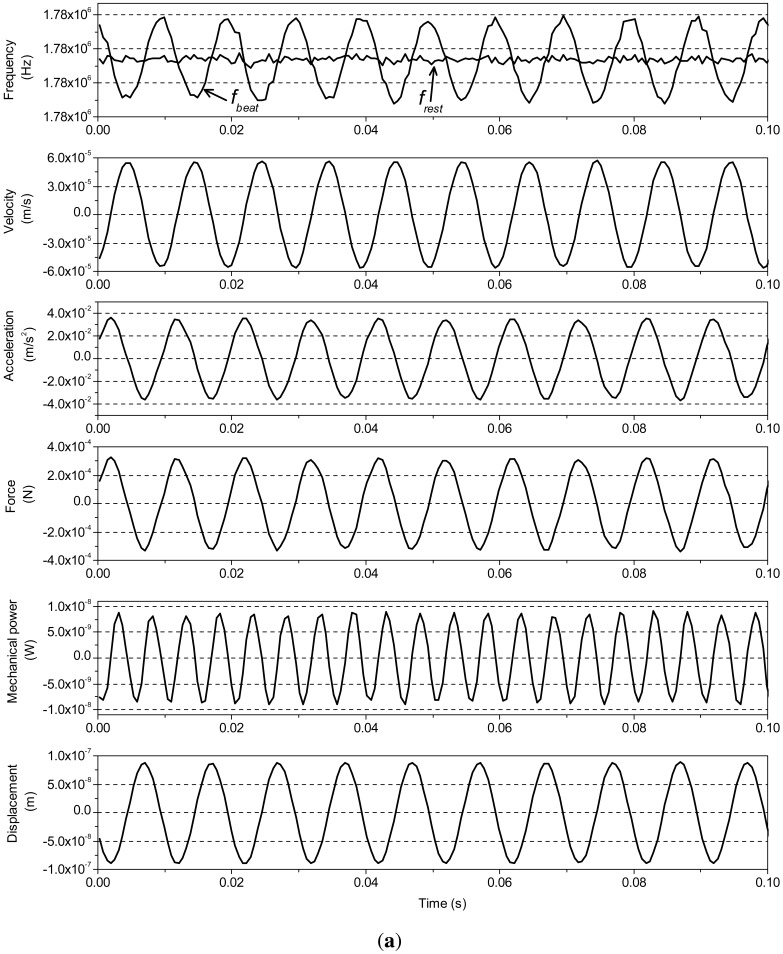

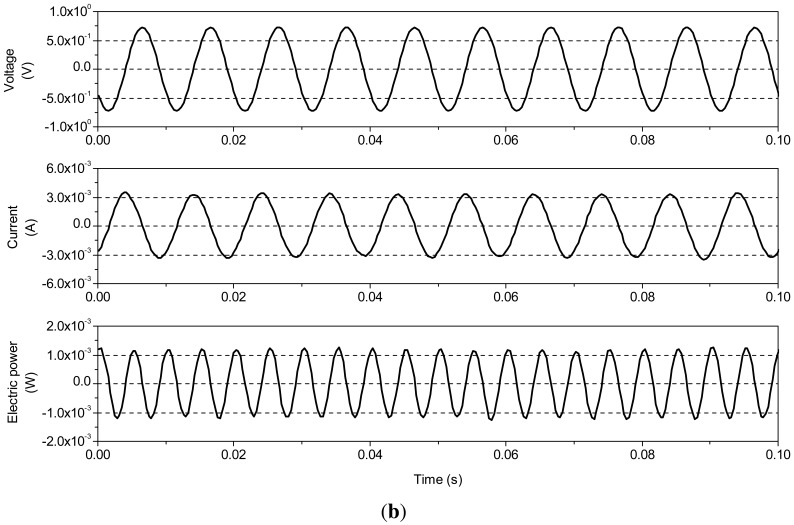
(**a**) The frequency of beat and rest laser beam, and mechanical quantities of PZT actuator P1; (**b**) The electrical quantities of PZT actuator P1.

All the actuators are discharged before tested. In the figures hereinafter shown, the data set obtained from the experiment of 0.1 s is shown. Since the driven signal and data processing for three actuators are same, Figure 3 only shows the mechanical and electrical quantities of P1, which are measured by using our experimental setup. Figure 3(a) shows the frequencies *f_rest_, f_beat_* and the other mechanical quantities, such us velocity, acceleration, force, mechanical power and displacement of actuator P1, calculated from the frequencies. The mechanical power is calculated as the product of force and velocity. Figure 3(b) shows the electrical quantities, voltage, current and electric power applied on P1. Usually, PZT actuator is considered as a capacitance load [20,21]. The phase difference between voltage and current is approximately 80.9° in Figure 3(b). This is because the phase difference is influenced by the back-emf, due to the hysteresis [22,23].

**Figure 4.**
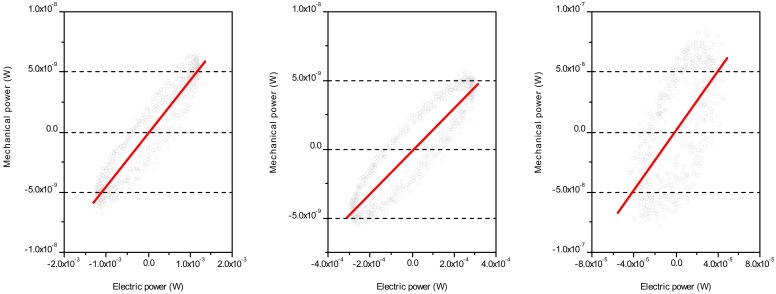
The instantaneous mechanical power changed against instantaneous electric power of P1, P2 and P3 from left to right.

The relationships between the instantaneous mechanical power *M_p_* and instantaneous electrical power *E_p_* of P1, P2 and P3 (from left-to-right) are shown in Figure 4. The regression lines of P1, P2 and P3 are *M_p_* = 4.4 × 10^−6^*E_p_* − 7.27 × 10^−11^, *M_p_* = 1.56 × 10^−5^*E_p_* + 5.53 × 10^−11^ and *M_p_* = 1.24 × 10^−3^*E_p_* + 1.4 × 10^−9^, respectively. The results show that small size of actuator can transform electric power into mechanical power more effectively. The efficiency of converting average power of electrical (*E_ave_*) power to mechanical (*M_ave_*) power of P1, P2 and P3 (*M_ave_/E_ave_*) are 4.8 × 10^−7^, 7.25 × 10^−6^ and 8.63 × 10^−5^, respectively. Most of the electric energy seems to be dissipated in the form of heat.

**Figure 5.**
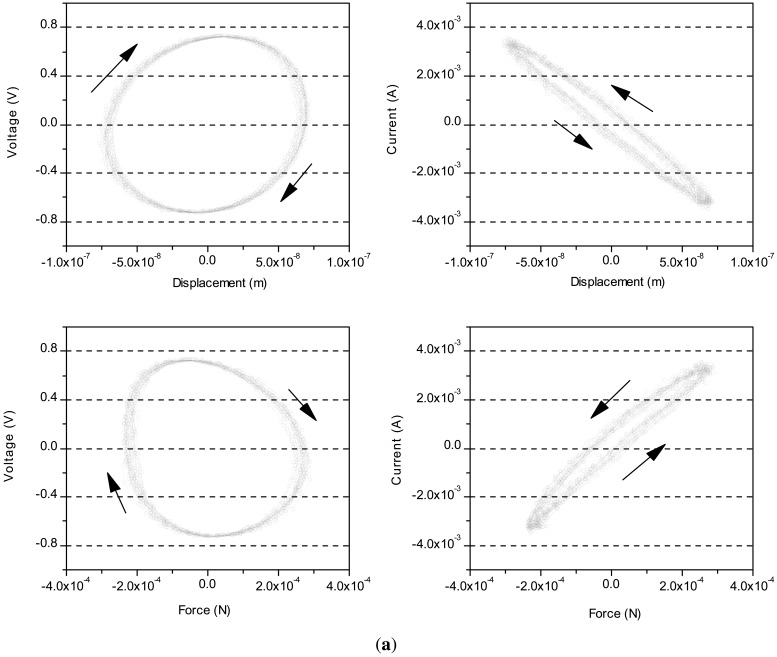

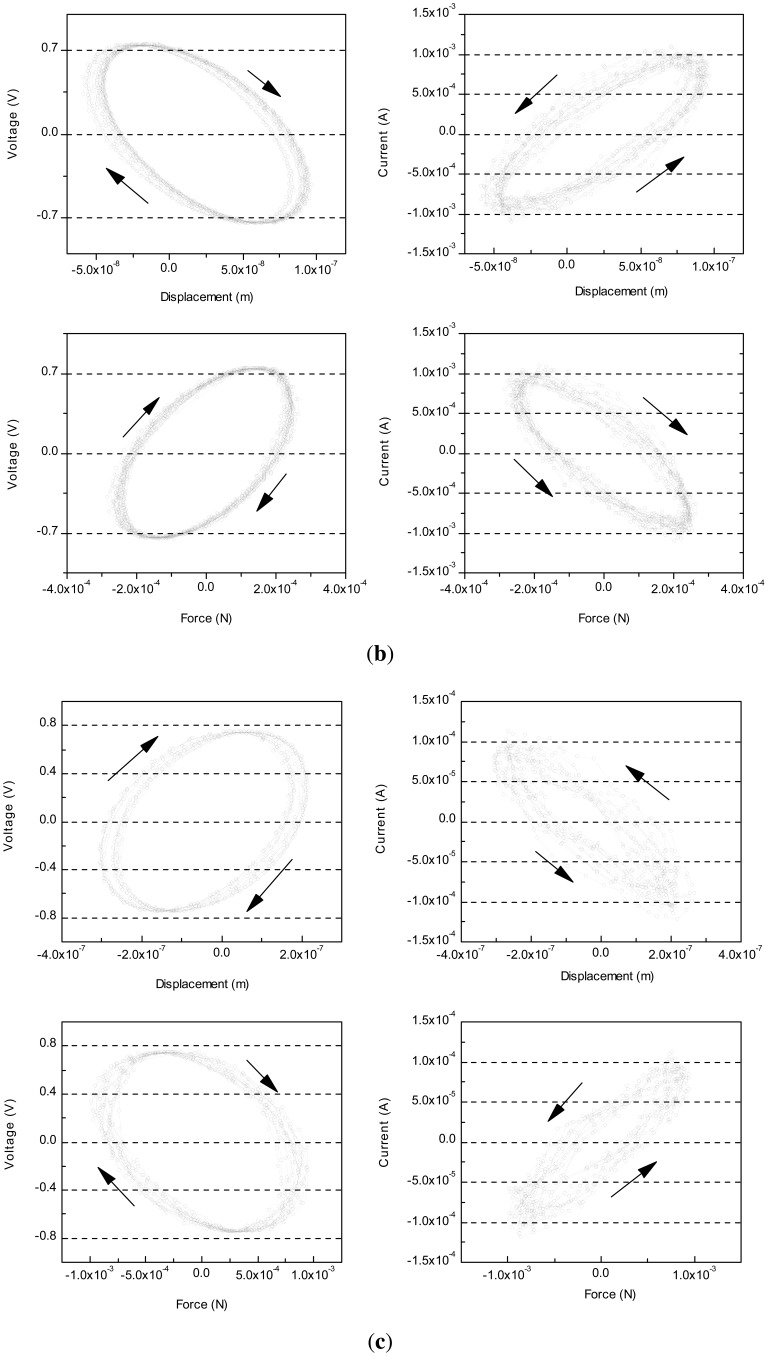
Voltage and current applied on the actuator, against displacement and force; (**a**) P1; (**b**) P2 and (**c**) P3.

Figure 5 shows a set of figures of voltage and current against displacement and force of the three actuators. These figures show that the hysteresis of the PZT actuators caused by voltage is larger than current. Particularly, the long length in the axial direction causes large hysteresis as shown in Figure 5(a,c). The high reproducibility of data indicates that the hysteresis loops are congruent due to the input variation within the same range.

**Figure 6.**
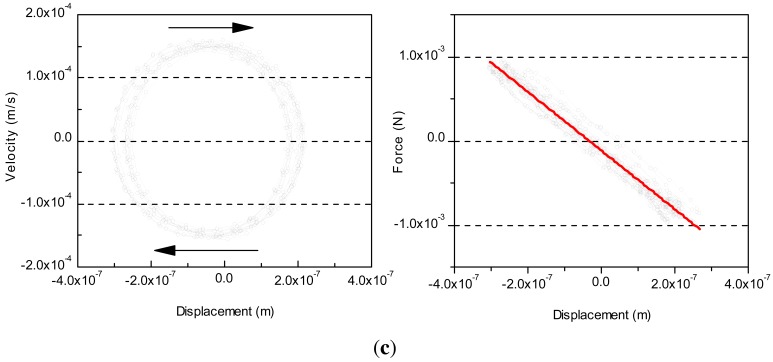

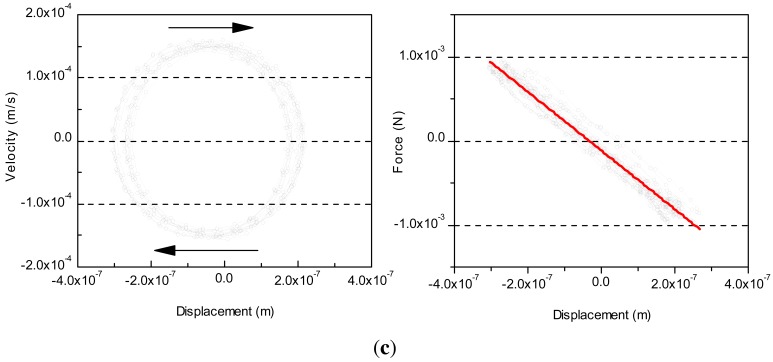
Velocity and force changed against displacement; (**a**) P1; (**b**) P2 and (**c**) P3.

Figure 6 shows the velocity and force against displacement of the actuators P1, P2 and P3. Ten periods of reciprocating motion are recorded during this experiment. The reproducibility of data is very high during reciprocating motion. The regression line of force *F* against displacement *x* is *F* = 3.02 × 10^−7^ − −3.56 × 10^3^*x* of P1, *F* = 7.35 × 10^−5^ − −3.54 × 10^3^*x* of P2 and *F* = −1.15 × 10^−4^ − −3.49 × 10^3^*x* of P3. From Figure 6, the actuators can be regarded as a spring with spring constant about 3.56 × 10^3^*N/m* of P1 (*k_p_*_1_), 3.54 × 10^3^*N/m* of P2 (*k_p_*_2_) and 3.49 × 10^3^*N/m* of P3 (*k_p_*_3_). In sinusoidal operation, the spring constant *k* can be concluded as: *k* = 4*π*^2^*m_eff_f*^2^ from manufacturer, where *f* is the operation frequency, *m_eff_* is the effective mass which relates to the loading and measuring point [7]. If the measuring point is assumed at the tip of PZT actuator, then *m_eff_* is considered as the mass of attachment, and *k* is equal to 3.58 × 10^3^*N/m*, which is 0.56% higher than *k_p_*_1_, 1.12% higher than *k_p_*_2_ and 2.58% higher than *k_p_*_3_. It may be because the measuring point where the acceleration is measured by interferometer is not equal to the tip of PZT actuator. In other words, this error may be caused by the mass between the tip of PZT actuator and the measuring point. The correlation coefficients of the fitting line in Figure 6 are higher than 0.9, indicating a very well linear relationship between force and displacement generated by the actuator.

Using the proposed method, the electro-mechanical characteristics of PZT actuators which are being driven can be measured easily and accurately. Force, which acts on the mass attached to the actuator under test, is measured as the inertial force of the mass itself, *i.e., F* = *Ma*. This is the most significant compared with other conventional methods. In this paper, only three sizes of stack piezo-stack actuators are tested. The developed method can be easily applied to evaluate the dynamic characteristics of different type of actuators and to evaluate the actuator driven by different types of excitation (e.g., changing the waveform, frequency or amplitude of the drive signal). This experiment is also able to calibrate actuators and estimate a mass whose inertial force can be measured by the calibration using the actuators.

The three dimensional measurement of the actuator can be realized by means of introducing three interferometers with three signal beams, which are not in the same plane and introduced to the CC attached to the actuator. Each signal beam has the sensitivity only for the component of the movement of the optical center (OC) of the CC of the signal beam's direction. If the gravity center (GC) of the attached mass coincides with the optical center (OC) of the CC, then the force component along each signal beam can be calculated as the product of the mass and the acceleration of the OC along the direction of each signal beam.

Precision measurements of the electrical and mechanical characteristics of the actuator should contribute to understanding the mechanism of the actuator and realizing more precision controllability of the actuator.

## 4. Conclusions

A precision method for evaluating the electrical and mechanical characteristics of actuators is proposed and demonstrated by evaluating the characteristics of three PZT actuators with difference size which are being driven. The main feature of the proposed method is that only two physical quantities are needed to be measured. One is Doppler frequency shift and the other is mass. Compared with above-mentioned method, force transducer, displacement transducer and known hold structure are not needed in this method. This method will be useful for better understanding the dynamic characteristics of PZT actuators during motion and for precisely controlling its position under dynamic conditions.

## Figures and Tables

**Table 1.** Specification of PZT actuators P1, P2 and P3.

**Name**	**Dimensions**	**Displacement(100V)**	**Capacitance**
P1	10 × 10 × 18 mm	15 *μ*m	6.59 *μ*F
P2	6.5 × 6.5 × 18 mm	15 *μ*m	1.6 *μ*F
P3	3.5 × 4.5 × 10 mm	9.1 *μ*m	0.18 *μ*F
